# Another Mastodon

**Published:** 1880-12

**Authors:** 


### ANOTHER MASTODON.

The remains of a large animal, probably a mastodon, were discovered in an old swamp near Hopestown, Ill., September 18. The tusks are nine feet long, twenty-six inches in circumference at the base, and weigh 175 pounds each. The lower jaw with teeth is well preserved. The teeth are perfect though somewhat worn. One weighs eight pounds, and is twenty-one inches in circumference. Several of the leg bones are in good condition. The thigh bone is two and a half feet long, and the tibia three feet. The ribs and back bone are in bad condition as the back of the animal was only three feet below the surface of the ground.

